# Proteomic investigation of effects of hydroxysafflor yellow A in oxidized low-density lipoprotein-induced endothelial injury

**DOI:** 10.1038/s41598-017-18069-4

**Published:** 2017-12-21

**Authors:** Feng Ye, Jianhe Wang, Wei Meng, Jingru Qian, Ming Jin

**Affiliations:** 1 Department of Pharmacology, Beijing Anzhen Hospital, Capital Medical University, Beijing Institute of Heart, Lung and Blood Vessel Disease, Beijing, 100029 China; 2 Guangdong Province Key Laboratory of Microbial Signals and Disease Control, State Key Laboratory for Conservation and Utilization of Subtropical Agro-Bioresources, College of Agriculture, South China Agricultural University, Guangzhou, 510642 China; 3 School of Biological Science, Nanyang Technological University, 60 Nanyang Drive, Singapore, 637551 Singapore

## Abstract

Oxidized low-density lipoprotein (ox-LDL)-induced vascular endothelial damage is a key event in early atherosclerosis. Safflower has been used to treat atherosclerotic heart disease in China for many years, but its molecular basis remains unclear. Hydroxysafflor yellow A (HSYA) is the main active ingredient of aqueous safflower extract. We identified the proteins involved in HSYA activity against ox-LDL-induced endothelial injury using isobaric tags for relative and absolute quantification-coupled two-dimensional liquid chromatography–tandem mass spectrometry. HSYA (1, 5, or 25 μM) alleviated ox-LDL-induced endothelial damage in a dose-dependent manner. We quantitated approximately 2700 protein species, of which 77 were differentially expressed following HSYA treatment. Most protein changes were related to structural molecules, metabolic enzymes, and proteins involved in signal transduction. Several differentially expressed proteins were further validated by western blot analysis. We also analysed the role of the mitochondrial membranous voltage-dependent anion-selective channel protein 2 (VDAC2) in HSYA treatment using small interfering RNA. VDAC2 functioned as a downstream anti-apoptosis effector during HSYA treatment of ox-LDL-induced endothelial impairment. These results further our understanding of the mechanisms responsible for the effects of HSYA.

## Introduction

Atherosclerosis is a common physiological disorder characterized by artery-wall thickening as a result of the invasion and accumulation of foam cells and proliferation of intima smooth muscle cells, which result in fibrofatty plaques^1,2^. Atherosclerosis is currently becoming a major health burden and one of the leading causes of death worldwide^3^. Extensive evidence suggests that endothelial function is impaired in animals and humans with hypercholesterolemia, and human epidemiologic and genetic studies strongly support lipoprotein as a causal risk factor for atherosclerosis^4–7^. Atherosclerosis is promoted by low-density lipoproteins (LDL), which are plasma proteins that carry cholesterol and triglycerides, and by inadequate removal of fats and cholesterol from macrophages by functional high-density lipoproteins. During hypercholesterolemia, LDL accumulates in the arterial wall and becomes oxidized (ox-LDL), thus impairing endothelial function and providing a key event in early atherosclerosis^2,6–9^. Ox-LDL causes damage to cultured endothelial cells^10^. The gaseous signalling molecule nitric oxide (NO), which contributes to vessel homeostasis, is decreased by the activity of ox-LDL^11^. Endothelium-derived NO mediates endothelial-dependent relaxation, and individuals with atherosclerosis, diabetes, or hypertension may present impaired^6,12,13^.

Safflower, the flower of *Carthamus tinctorius* L., has been extensively used in Traditional Chinese Medicine for treating cardiovascular and gynaecological diseases. The active component of aqueous safflower extract, safflor yellow, used in the treatment of coronary heart disease, usually caused by atherosclerosis. Furthermore, hydroxysafflor yellow A (HSYA), the main active ingredient of safflor yellow, is approved for treating cardiac patients in China^14,15^. The Akt-dependent autophagy pathway has been suggested to contribute to HSYA-afforded neuroprotection^15^. We previously showed that HSYA alleviated lipopolysaccharide-induced endothelial injury and attenuated levels of inflammatory factors induced by platelet activating factor or lipopolysaccharide^16–18^. However the molecular mechanisms whereby HSYA protects the endothelium remain obscure.

The present study aimed to identify the proteins associated with the regulation of early atherosclerosis and the effects of HSYA. For the comparison of differentially expressed proteins, the availability of high-throughput methods allow for highly accurate and precise analyses expression profile changes. In particular, isobaric tags for relative and absolute quantification (iTRAQ) technology has gained popularity in quantitative proteomics because of its accurate quantitation and high sensitivity^19^. We screened a human umbilical vein cell line using iTRAQ-coupled two-dimensional liquid chromatography–tandem mass spectrometry (2D LC-MS/MS). This systematic proteomic approach allowed the identification of novel proteins associated with the mechanisms of HSYA action.

## Results

### Malondialdehyde (MDA) and NO assays

Human umbilical vein endothelial cells (EA.hy926) were pretreated with or without HSYA (1, 5, 25 μM) for 30 min, and then incubated with or without ox-LDL (80 μg/ml) for 24 h. The samples were collected for MDA and NO assays. MDA is a biomarker of oxidative stress levels. The mean (±standard error) concentration of MDA in the control group was 2.993 ± 0.258 nmol/ml. Pretreatment with ox-LDL (80 μg/ml) for 24 h significantly increased the MDA content in cultured endothelial cells (Table 1), while HSYA (1, 5, or 25 μM) decreased MDA in a dose-dependent manner. Previous study has reported that aspirin attenuated endothelial damage in the animal treated with LDL^6^. Our results demonstrated that ox-LDL-injured endothelial cells pretreated with aspirin (5 mM), as a positive control, also inhibited the increase in MDA levels.

**Table 1:** Effect of HSYA on the medium levels of MDA and NO (n = 4 per group).

Name	Con	OX-LDL(80 μg/ml)	+HSYA (1 μM)	+HSYA (5 μM)	+HSYA(25 μM)	Con + HSYA(25 μM)	Con + Aspirin(5 mM)	OX-LDL + Aspirin (5 mM)
MDA (nmol/ml)	2.993 ± 0.258	7.02 ± 0.42^**^	5.988 ± 0.227	4.915 ± 0.332^##^	5.278 ± 0.39^#^	3.125 ± 0.22	2.878 ± 0.31	5.12 ± 0.21^#^
NO (μM)	53.25 ± 3.59	32.75 ± 2.02^*^	36.75 ± 2.29	45.25 ± 2.93^#^	42.25 ± 2.75^#^	52.00 ± 3.24	55.25 ± 3.79	42.00 ± 3.41

Data expressed as means ± S.E.M. Statistical analyses were performed using one-way analysis of variance (ANOVA) with Student–Newman–Keuls post hoc test. The acceptable level of significance was ^*^ *P* < 0.05, ^**^ *P* < 0.01 vs Con group, ^#^ *P* < 0.05, ^##^ *P* < 0.01 vs OX-LDL group.

The NO content in the control group was 53.25 ± 3.59 μM. However, NO levels were decreased in the ox-LDL group compared with the control group, while HSYA (1, 5, or 25 μM) significantly increased NO levels in a dose-dependent manner. Pretreatment with aspirin (5 mM) also alleviated the decrease in NO content (Table 1).

### iTRAQ analysis of differentially expressed proteins between HSYA-treated and ox-LDL-injured endothelial cells

**Figure 1:**
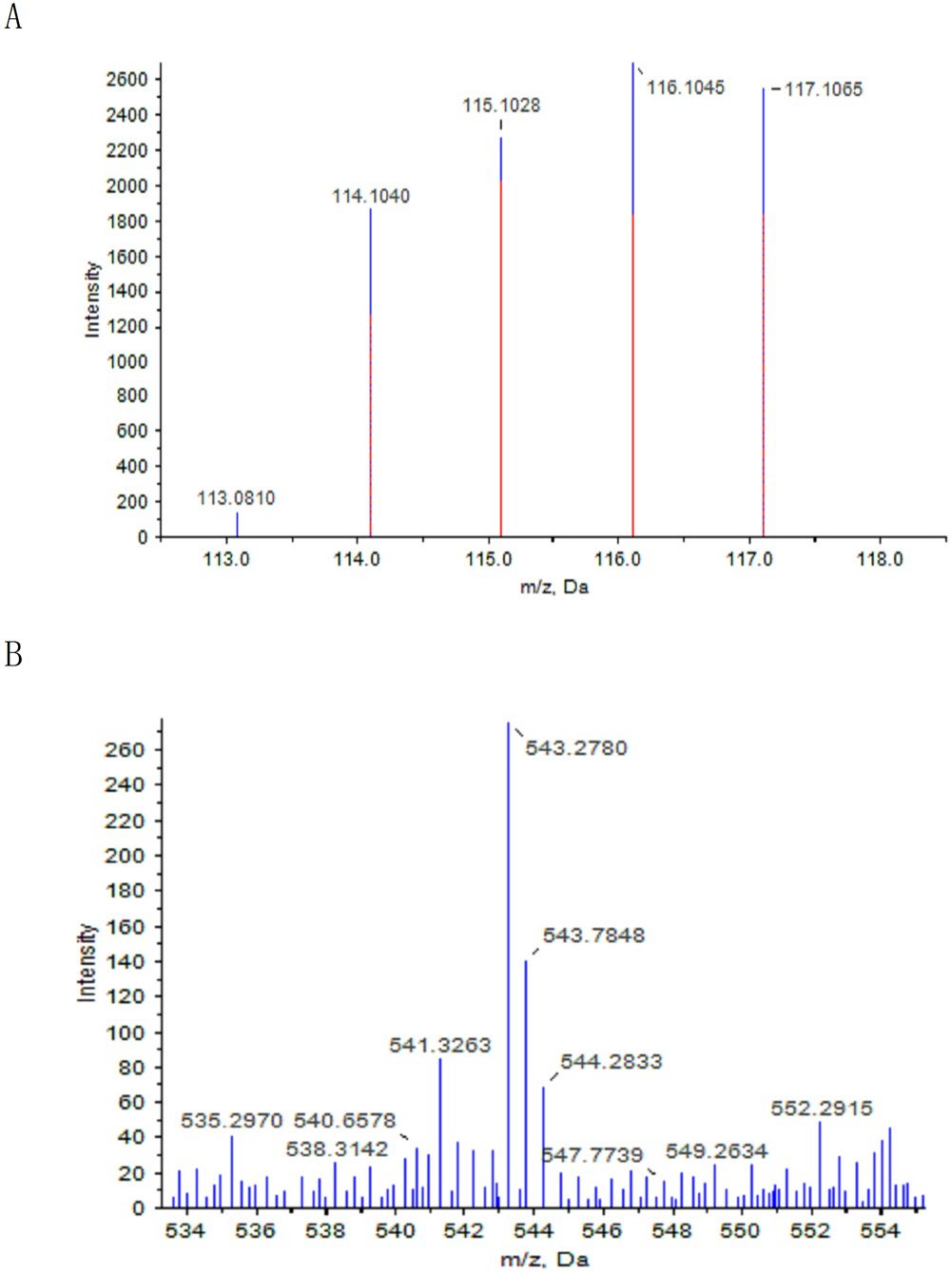
iTRAQ proteomics approach was used to identify differentially expressed proteins between HSYA-treated and ox-LDL-injured endothelial cells. A representative MS/MS spectrum showing the peptides from VDAC2 (peptide sequence: YQLDPTASISAK). Ox-LDL-injured endothelial cells (ox-LDL injury) were labeled with iTRAQ 115 tags, and HSYA-treated ox-LDL-injured endothelial cells (HSYA treatment) were labeled with iTRAQ 116 tags. The assignments are as follows (**A**) ratio; (**B**) MS/MS).

**Figure 2:**
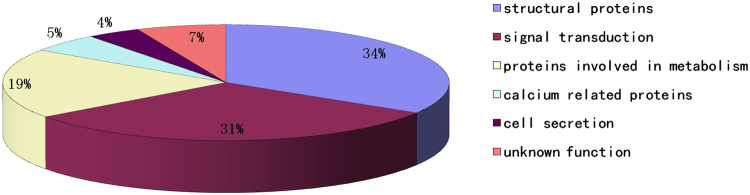
Distribution of all proteins identified after iTRAQ labeling and mass spectrometry into different functional categories.

EA.hy926 cells were digested with trypsin overnight at 37 °C. Peptides from HSYA-treated and ox-LDL-injured cells were labeled at their free amine sites using isobaric mass tag labels, mixed, and analysed by reverse phase LC-MS/MS. Upon collision-induced dissociation, the parent peptides were broken up and the associated isobaric mass tags were released. Dissociation of the parent peptide yielded a characteristic mass-fragmentation pattern that allowed the parent protein to be identified by comparing the fragmentation fingerprint to theoretical protein digests. Comparative peptide data could be obtained for multiple proteins from one experiment, thus reducing variability due to peptide measurements between samples. We quantified 2743 proteins by iTRAQ analysis, and identified 77 differentially expressed proteins after testing for multiple comparisons from the quantitative information (Supplementary Table S1). The representative MS/MS of VDAC2 is shown in Fig. 1. Proteins identified as differentially expressed had to demonstrate fold changes >1.4 by Protein Quant assay. According to this criterion, 30 proteins were up-regulated and 47 were down-regulated following HSYA treatment. These differential proteins were classified into six groups according to their cellular functions (Fig. 2): cellular structure (including cytoskeletal proteins, molecular chaperones) (n = 26), cell signalling pathways (including transcription factors) (n = 24), cellular metabolism (n = 15), cell secretion (n = 3), calcium function (n = 4), and unknown function (n = 5). All six groups included some proteins with the same cellular function that were down- and up-regulated in the experiments (e.g., 11 of 24 signalling proteins were up-regulated and 13 were down-regulated). Some proteins (e.g., NIPS1, VDAC2, histone H1.2, AIFM1) increased following treatment with HSYA compared with the ox-LDL-damage group, while other proteins (e.g., GSTP1, HMG-17, PSA7) decreased (Supplementary Table S1). Interestingly, expression levels of VDAC2 and VDAC1 were simultaneously increased.

### Validation of differentially expressed proteins by western blot analysis

**Figure 3:**
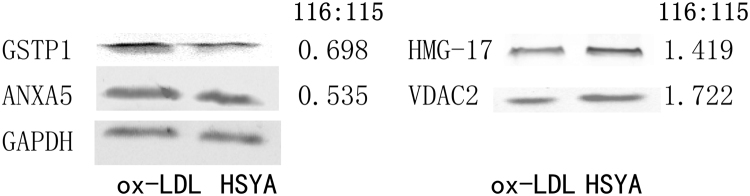
Western blot analysis of protein levels of (1) GSTP1; (2) HMG-17; (3) ANXA5; (4) VDAC2; and (5) GAPDH in ox-LDL injury and HSYA treatment. GAPDH in the cell lysate served as a loading control. Four independent experiments were performed. The 116:115 is iTRAQ ratio of different protein expression level in HSYA treatment relative to ox-LDL injury. Full-length blots are presented in Supplementary Figure 1.

We verified the significance changes in protein level quantitated by iTRAQ-coupled 2D LC-MS/MS using western blot analyses of five proteins (GSTP1, HMG-17, ANXA5, VDAC2, and GAPDH). All western blot results were consistent with the respective LC-MS/MS analyses. Similar to the iTRAQ analysis, the levels of GSTP1 and ANXA5 were lower in the HSYA-treated compared with the ox-LDL-injured cells (Fig. 3). Quantification of HMG-17 and VDAC2 showed increased levels following HSYA treatment, compared with the control ox-LDL-injured cells. VDAC2 expression levels were significantly increased by HSYA compared with untreated ox-LDL-injured cells, whereas glyceraldehyde 3-phosphate dehydrogenase (GAPDH) expression levels remained unchanged. Significant changes based on four independent experiments were analysed by infrared imaging and showed consistent results with the respective LC-MS/MS analyses. These results confirmed the validity of the iTRAQ results at the protein level.

### Association between VDAC2 and HSYA treatment

**Figure 4:**
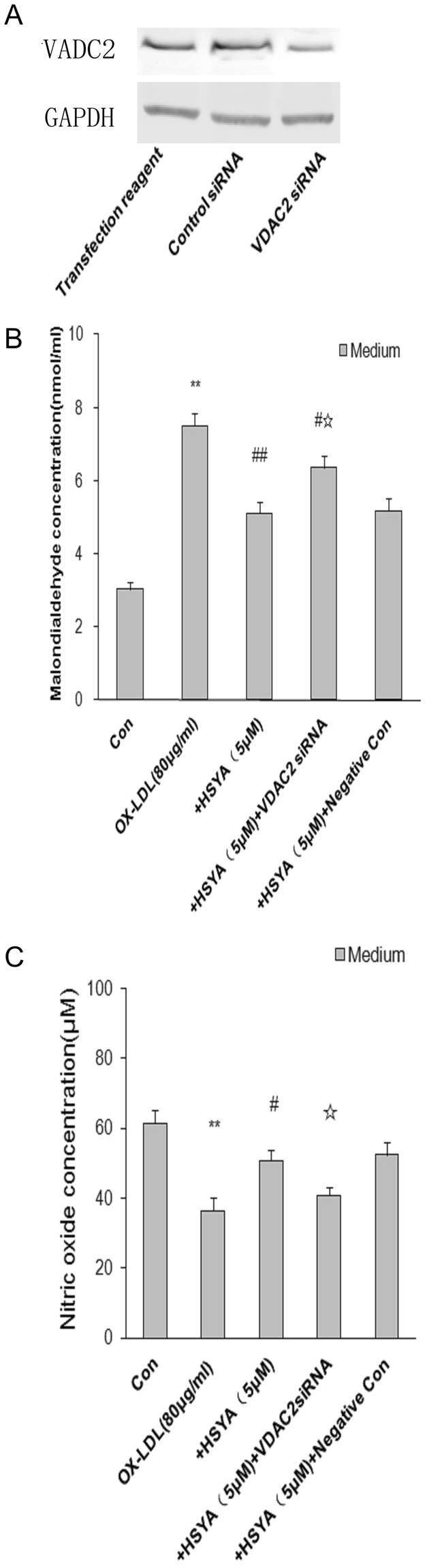
(**A**) Western blot analysis showed that transfection of EA.hy926 cells with VDAC2 siRNA significantly reduced VDAC2 protein levels, whereas VDAC2 protein expressions were not significantly suppressed by control siRNA and transfection medium(sc-36868). GAPDH in the cell lysate served as a loading control. Cells were treated with siRNA transfection medium(sc-36868) only. Four independent experiments were performed. Full-length blots are presented in Supplementary Figure 2. (**B,C**) Furthermore, (**B**) and (**C**) showed that transfection of EA.hy926 cells with VDAC2 siRNA could change both MDA and NO levels in the conditioned medium. Values are mean ± S.E.M, n = 4 per group. Statistical analyses were performed using one-way analysis of variance (ANOVA) with Student–Newman–Keuls post hoc test. ^*^ *P* < 0.05 considered as statistically significant. ^*^ *P* < 0.05, ^**^ *P* < 0.01 vs Con group, ^#^ *P* < 0.05, ^##^ *P* < 0.01 vs OX-LDL group, ^☆^ *P* < 0.05 vs HSYA group.

We examined the functional role of voltage-dependent anion-selective channel protein 2 (VDAC2) that was up-regulation in HSYA treatment by transfecting EA.hy926 cells with VDAC2 small interfering RNA (siRNA). VDAC2 siRNA, but not negative control siRNA, significantly decreased VDAC2 protein levels, as demonstrated by western blotting (Fig. 4A). Futhermore, we evaluated the effect of VDAC2 siRNA on MDA and observed a significant increase in the MDA content in the HSYA-treated cells. However, there was no significant difference in MDA levels between HSYA-treated groups and HSYA-treated groups pretreated with VDAC2 negative control siRNA (Fig. 4B). In addition, VDAC2 siRNA reduced NO levels in the HSYA-treatment group. There were no significant differences in NO levels between HSYA-treated groups and HSYA-treated groups pretreated with VDAC2 negative control siRNA (Fig. 4C).

### Terminal deoxynucleotidyl transferase dUTP nick end labeling (TUNEL)/4′, 6-diamidino-2-phenylindole (DAPI) and Annexin V/propidium iodide (PI) staining assays

**Figure 5:**
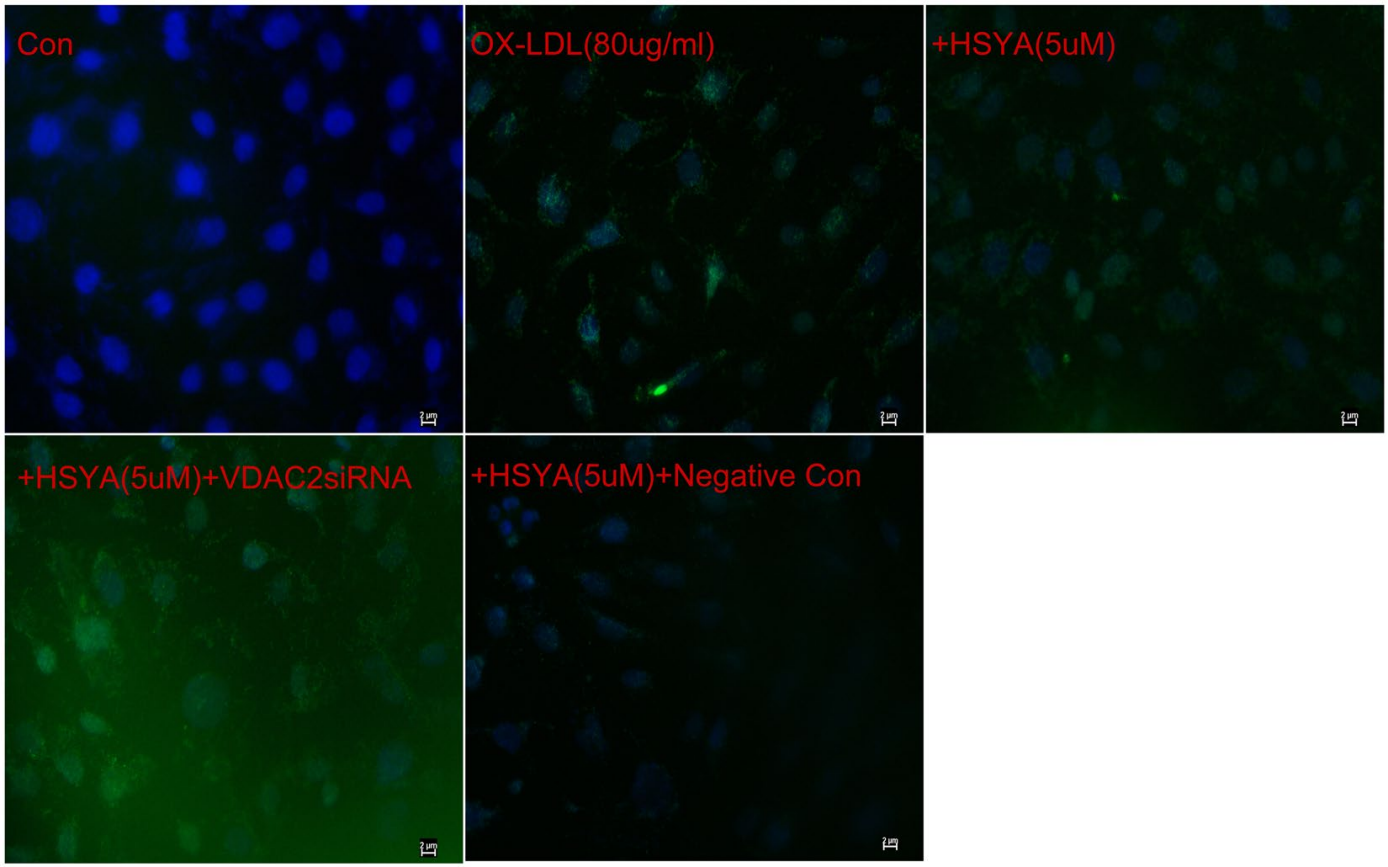
TUNEL/DAPI double staining assay. Analysis of apoptosis by TUNEL staining in human endothelial cell was performed under different conditions. Counter staining was performed using DAPI nuclear staining and pictures were taken using Merged filters (DAPI and FITC (fluorescein isothiocyanate)). Results are shown for healthy control endothelial cells (Con), OX-LDL endothelial cells, OX-LDL endothelial cells treated with HSYA and HSYA endothelial cells transfected with VDAC2 siRNA, and VDAC2 siRNA negative control respectively. OX-LDL endothelial cells show smaller nuclei on the DAPI stain and increased TUNEL staining in the cytoplasm of OX-LDL endothelial cells compared to healthy controls, reflecting a significant increase of apoptosis. Following treatment with HSYA, a decrease of apoptosis was observed, whereas after transfection with VDAC2 siRNA, increase or decrease in apoptosis was observed in comparison with OX-LDL endothelial cells treated with HSYA. Furthermore, no difference was observed after transfection with VDAC2 negative control siRNA compared to OX-LDL endothelial cells treated with HSYA. Three to four independent experiments were performed. Figure 5 showed that transfection of EA.hy926 cells with VDAC2 siRNA could inhibit anti-apoptosis effect of HSYA by TUNEL**/**DAPI staining. Scale bar = 2 μm.

**Figure 6:**
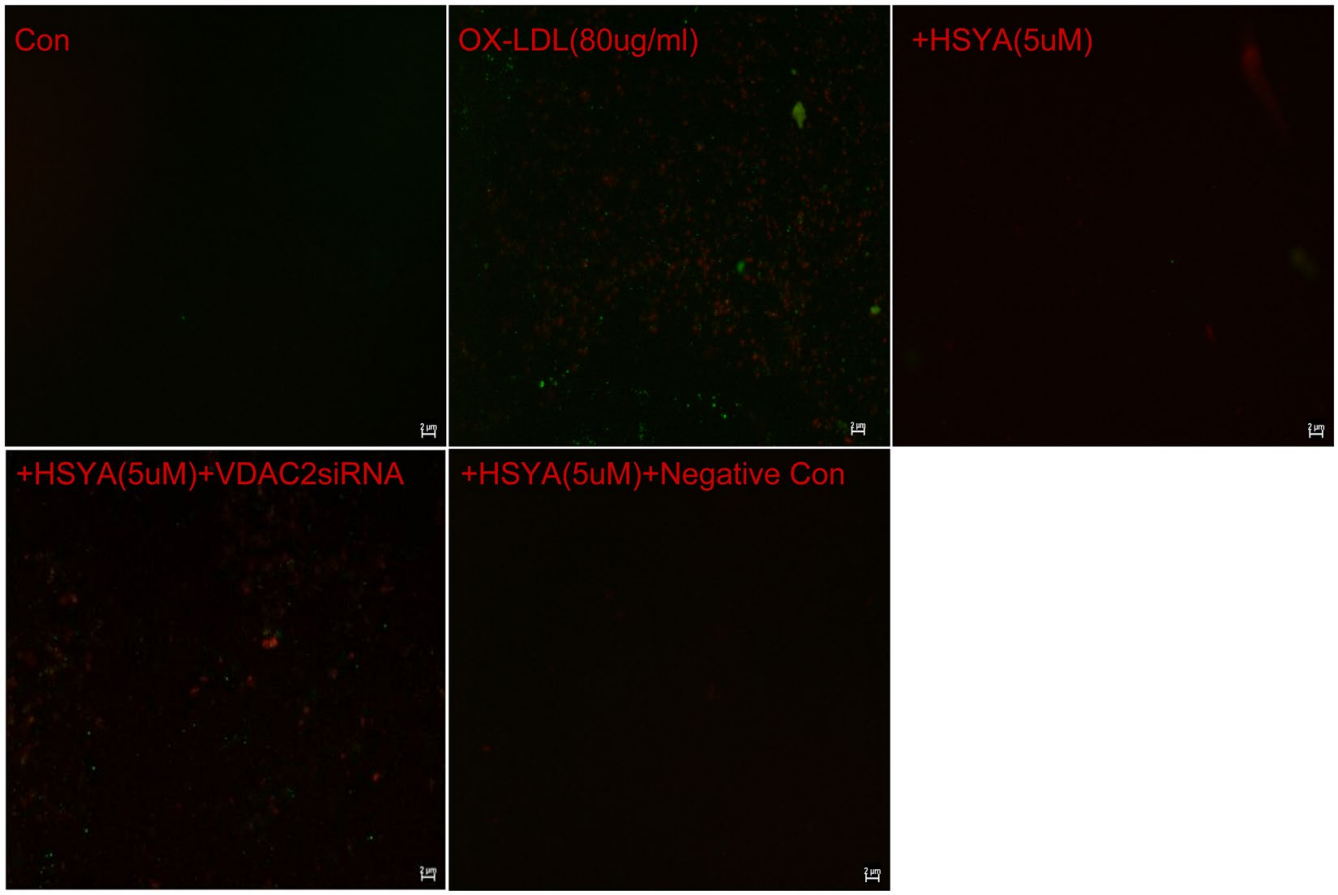
Annexin V/PI double staining assay. We evaluated the presence of apoptosis by Annexin V/PI staining assay in cultured endothelial cell under different conditions. Counter staining was performed using PI staining and pictures were taken using Merged filters (PI and FITC). Results are shown for healthy control endothelial cells (Con), OX-LDL endothelial cells, OX-LDL endothelial cells treated with HSYA and HSYA endothelial cells transfected with VDAC2 siRNA, VDAC2 siRNA negative control respectively. OX-LDL endothelial cells show significantly increased Annexin V and PI staining in OX-LDL endothelial cells compared to healthy controls, reflecting an increase of apoptosis. Following treated with HSYA, a decrease of apoptosis was observed, whereas after transfection with VDAC2 siRNA, increase or decrease in apoptosis was observed in comparison with OX-LDL endothelial cells treated with HSYA. Additional, no difference was observed after transfection with VDAC2 negative control siRNA compared to OX-LDL endothelial cells treated with HSYA. Three to four independent experiments were performed. Figure 6 showed that transfection of EA.hy926 cells with VDAC2 siRNA could inhibit anti-apoptosis effect of HSYA by Annexin V/PI staining. Scale bar = 2 μm.

Apoptosis is an important mechanism in endothelial damage. We studied the association between HSYA and apoptosis by TUNEL/DAPI and Annexin V-fluorescein isothiocyanate (FITC)/PI assays, and obtained images using merged filters. Apoptosis was largely absent in healthy human endothelial cells (Figs 5 and 6, above left). However, treatment with ox-LDL (80 μg/ml) resulted in increased apoptosis which was evident by an accumulation of fluorescence (green and red), while apoptosis was significantly reduced following treatment with HSYA (5 μM) in cultured human umbilical vein cells. Cell nuclei were stained with DAPI (blue). Further evaluation of VDAC2 effect on apoptosis revealed that VDAC2 siRNA partly blocks the anti-apoptosis effect of HSYA, as shown by accumulated green and red fluorescence (Figs 5 and 6, below left). However, there was no significant difference in apoptosis between HSYA-treated groups with and without pretreatment with VDAC2 negative control siRNA. These findings suggest that the anti-apoptosis effect of HSYA was partially mediated by VDAC2. The results were also consistent with the MDA and NO results (Fig. 4B,C).

## Discussion

Atherosclerosis is a chronic disease initiated by inflammatory events or processes in endothelial cells of vessel walls, associated with the retention of LDL particles^20^. Although the atherosclerotic process is currently not clearly understood, a substantial body of evidence has implicated ox-LDL-induced endothelial injury or dysfunction as a key event in the early phase of atherosclerosis, and thus in the pathogenesis of diverse diseases^2,8,9^. In this study, we used an iTRAQ proteomic approach to identify proteins regulated by HSYA, which may affect early atherosclerosis. The results identified 77 proteins with altered expression levels following HSYA treatment of ox-LDL-injured endothelial cells. Furthermore, we demonstrated an association between VDAC2 and HSYA treatment of ox-LDL-induced endothelial impairment, and showed that VDAC2 siRNA markedly inhibits the reduction of ox-LDL-induced endothelial injury by HSYA.

iTRAQ has recently gained popularity for diverse research applications such as posttranslational modifications and biomarker discovery. Using this high-throughput proteomic technology, we screened 77 proteins altered expression levels following treatment with HSYA in endothelial cells. Categorization of the differentially expressed proteins into their cellular functional groups, showed that most affected proteins (65%) were associated with signal transduction and structural molecules. These results confirm that iTRAQ is a powerful tool for the detection of low-abundance proteins with important roles in cell signalling pathways, to its high sensitivity, accurate quantitation, and high reproducibility^19^.

Atherosclerosis affects arterial blood vessels but may remain asymptomatic for decades^1^. Although the atherosclerotic process is not fully understood, it is thought to be initiated by inflammation due to association with retained LDL particles in the endothelial cells of the vessel wall^20^. The particles are oxidized by free radicals^21^, resulting in ox-LDL particles that trigger a cascade of inflammatory signal responses, such as CD40/CD40L^22^, and increase the activity and expression of matrix metalloproteinase-1 and -3^23^ in coronary artery endothelial cells. Ox-LDL enhances the expression of pro-inflammatory genes, leading to dysfunction of vascular endothelial cells and monocyte recruitment into the vessel wall^24^. Endothelial dysfunction is widely considered as the earliest event in atherosclerotic diseases and a major pathophysiological mechanism that leads to coronary artery disease and other atherosclerotic diseases^1^. Thus ox-LDL has been highly implicated as a critical factor in atherogenesis^7,8^.

Protein kinase Cβ (PKCβ) silence for vascular smooth muscle cells significantly suppresses ox-LDL-induced apoptosis, and PKCβ activation hinges on the reactive oxygen produced by ox-LDL. PKCβ participates in the apoptotic pathway via IRE1a/JNK pathway^25,26^. Previous study reported the relevance between B-cell lymphoma-2 (Bcl-2) protein and apoptosis in the atherosclerotic process^27^. Besides Bcl-2, Bax expression protects against ox-LDL-induced apoptosis as well^25,28^. Granulocyte–macrophage colony stimulating factor (GM–CSF) also takes part in the pathogenesis of atherosclerosis. It generates IL-23, which improves apoptosis by augmenting proteasome-dependent degradation of Bcl-2 in the LDL-driven atherosclerosis^29^. Retinol binding protein 4 (RBP4) promotes the pathogenesis of atherosclerosis. It has been connected to cardiovascular system disease. RBP4 enhances superoxide production in the dose-dependent style in the human aortic endothelial cells^25,30^. Ox-LDL binding meanwhile irritates superoxide dismutase (SOD) expression. SOD declines apoptotic impact on the dose- and time-dependent ways^25,31^. Homocysteine induces endothelial function disorder and atherosclerosis through reactive oxygen production. It increases mitochondrial dysfunction in the endotheliocytes. Homocysteine exerts its effects via multiple molecules (3-nitrotyrosine (3-NT), nuclear factor-κB (NF-κB), mitochondrial transcription factors A (Tfam), nuclear respiratory factor-1(NRF-1), and cytochrome c oxidase subunit III)^25^. Angiotensin II is involved in the progress of artheroclerosis and increases endothelial xanthine oxidase^32^. Angiotensin II also enhances CD40/CD40L activated in the angiocellulars^33^. It was reported that CD40/CD40L played a key role in the atherosclerotic process^25^. In addition, nod-like receptor pyrin domain-containing 3 (NLRP3) inflammasome is involved in the chronic inflammation under atherogenesis in the vascular walls^34^.

HSYA, a main component of safflor yellow, protects endothelial cells against LPS-induced impairment by inhibiting inflammatory factor levels via the NF-κB pathway^16,18^. It also attenuates LPS-induced NLRP3 inflammasome activation via binding to xanthine oxidase in the RAW264.7 macrophage cell line^35^. Additionally, HSYA was shown to decrease the levels of platelet activating factor-induced inflammatory molecules^17^. However, the molecular regulatory network of HSYA is still unclear. In this study, we investigated the effects of HSYA on human endothelial cell damage and explored its possible mechanisms using iTRAQ coupled with verification assays by various molecular biology techniques. Ox-LDL significantly decreased NO levels, and both the MDA and NO analysis confirmed that HSYA reduced endothelial injury in a dose-dependent manner. The MDA results were consistent with those from previous studies^10^. iTRAQ analysis demonstrated that HSYA triggers a cascade of signalling pathways involving a complex regulatory network. There was a complicated relationship between the different proteins in terms of their up- or down-regulation.

Interestingly, many of the signalling proteins identified in the current study were related to apoptosis. e.g. AIFM1 can function both as regulator of apoptosis and as an NADH oxidoreductase^36^, while KDIS modulates stress-induced apoptosis of melanoma cells via regulation of the MEK/ERK signalling pathway^37^. Both VDAC2 and VDAC1 are involved in cell apoptosis and volume regulation, as well as the formation of the permeability transition pore complex (PTPC) responsible for the release of mitochondrial products that triggers apoptosis^38,39^. PSA7 also plays an important role in the regulation of cell apoptosis, proliferation, and transcriptional regulation^40^, whiles PRKCE participates in the immune response and regulation of apoptosis^41^. To the best of our knowledge, this study provides the first evidence for the involvement of these proteins in the action of HSYA against ox-LDL-induced endothelial injury.

VDACs are porin ion channels located on the outer mitochondrial membrane^38^. Three mammalian isoforms,VDAC1, VDAC2, and VDAC3, with different physiological roles, are PTPC components of the mitochondrial inner and outer membranes. The current iTRAQ data showed that VDAC2 and VDAC1 were markedly increased in HSYA-treated ox-LDL-injured endothelial cells. And VDAC2 showed larger changes in protein expression than VDAC1. Formation of the PTPC, the subsequent dissipation of mitochondrial potential, and the release of cytochrome c are known to be critical events in the early stages of apoptosis^19,39^. VDACs have been suggested to participate in programmed cell death^39,42,43^. Some studies have indicated that VDAC2 may play an important role in maintaining cell viability^42^, while another report demonstrated that VDAC2 significantly alleviated apoptosis mediated by Bak^43^. The current results of TUNEL/DAPI and Annexin V/PI assays showed that HSYA markedly ameliorated endothelial cell apoptosis via the VDAC2 pathway, and suggested that VDAC2 may play a key role in the regulation of apoptosis. HSYA treatment also significantly increased NO levels following ox-LDL-induced endothelial injury. Despite these results, the molecular mechanisms involved in VDAC2 up-regulation have not been fully elucidated, and further studies are needed to understand the downstream molecular events of HSYA.

Autophagy is a process of intracellular bulk degradation that leads to regulated cell destruction. It is considered as an adaptive response to stress, promoting survival^44,45^. Autophagy is related to cardiovascular diseases because it is triggered by oxidized lipoprotein, oxidative stress, inflammation, hypoxia, and ER stress which are all involved to atherogenesis^25^. However, the role of autophagy in atherosclerosis remains obscure. 7-oxysterols, an important toxic components in ox-LDL, leads to lysosomal membrane permeabilization (LMP) and cell death^46^. Autophagy counters LMP and cell death in the atherosclerosis abduced by 7-oxysterols. Interestingly, the present iTRAQ data showed that most HSYA-induced protein changes were down-regulated as observed in autophagy thereby enabling the orderly degradation and recycling of cellular components^45,47^. The Akt-dependent autophagy pathway was previously shown to contribute to the HSYA-afforded neuroprotection^15^. Inhibition of autophagy via silencing ATG5 and others autophagy mediators enhances apoptosis^25^. We therefore speculated that HSYA may reduce cellular components to promote survival by maintaining cellular energy levels in ox-LDL-induced endothelial injury. However, the role, if any, of autophagy in endothelial impairment remains unclear, and further studies are needed to address this issue. Overall, our results suggest that HSYA may exert its effects against ox-LDL-induced endothelial injury via multiple mechanisms. To our best knowledge, most of altered proteins were first discovered in HSYA treatment. These data provide new targets for future investigations with respect to HSYA.

The complex aetiology of atherosclerosis requires full understanding of the molecular events occurring during endothelial dysfunction. In this study, we demonstrated that HSYA induced changes in the expression profiles of 77 protein species in the endothelial cell proteome, using iTRAQ analysis. We also identified novel proteins and demonstrated an association between VDAC2 expression and endothelial damage. We showed that HSYA treatment could significantly improve ox-LDL-induced human endothelial injury, partially via the anti-apoptosis effect of VDAC2. This survey of protein expression provides valuable information to aid the broader understanding of early-stage atherosclerosis, and provides new targets for future investigations with respect to atherosclerosis.

## Materials and Methods

### Reagents

HSYA was isolated and purified from the aqueous extract of *C. tinctorius* L. by macroporous resin-gel column chromatography, as described previously^14^. The molecular weight of HSYA is 612. HSYA was analysed using a high-performance liquid chromatography system (Shimadzu, Kyoto, Japan). iTRAQ kits were purchased from Applied Biosystems (Waltham, MA, USA), the 2-D Quant Kit from GE Healthcare (Pittsburgh, PA, USA), sequence-grade modified trypsin from Promega (Madison, WI, USA), and human ox-LDL (BT-910) from Alfa Aesar (Heysham, Lancashire, UK). Aspirin was the product of Sigma-Aldrich (St. Louis, MO, USA). Bromophenol blue, tetramethylethylenediamine, Coomassie brilliant blue G-250, Bis, low-molecular weight marker, nitrocellulose membrane, Tris-base, and the Bradford method protein assay kit were purchased from Bio-Rad (Hercules, CA, USA). VDAC-2 polyclonal antibody was purchased from Cell Signaling Technology (Danvers, MA, USA), and GAPDH mouse monoclonal antibody was from Beyotime Biotechnology (Jiangsu, China). Other polyclonal and monoclonal antibodies and siRNA reagents were purchased from Santa Cruz Biotechnology (Santa Cruz, CA, USA), with the secondary antibodies obtained from LI-COR Biosciences (Lincoln, NE, USA). NO and MDA assay kits were purchased from Nanjing Jiancheng Bioengineering Institute (Nanjing, China).

### Cell culture

The human umbilical vein cell line EA.hy926 (cell bank, Shanghai Institute for Biological Sciences, Chinese Academy of Sciences) was cultured in DMEM medium (Thermo Fisher Scientific, Waltham, MA, USA) supplemented with 10% fetal bovine serum (Thermo Fisher Scientific) and 1% penicillin-streptomycin (Thermo Fisher Scientific) at 37 °C in a humidified atmosphere of 5% CO_2_/95% air.

### Groups

The experiments comprised three sections. For section 1, EA.hy926 cells were divided into eight groups as follows: cells were pretreated with or without HSYA (1, 5, 25 μM) for 30 min and then incubated with or without ox-LDL (80 μg/ml) for 24 h. In parallel experiments, other groups of cells were pretreated with aspirin (5 mM) for 30 min and then incubated with or without ox-LDL (80 μg/ml) for 24 h. Conditioned medium was collected for NO and MDA measurements (Table 1). Section 2 comprised two groups: cells were pretreated with or without HSYA (5 μM) for 30 min, and then incubated with ox-LDL (80 μg/ml) for 24 h. Cells were then harvested for both iTRAQ experiments and validation (Fig. 3, Supplementary Table S1). Section 3 comprised five groups: after cells reached confluence, they were transfected with or without VDAC2 siRNA, then pretreated with or without HSYA (5 μM) for 30 min, followed by incubation with or without ox-LDL (80 μg/ml) for 24 h. Other group of cells were transfected with VDAC2 negative control siRNA, then pretreated with HSYA (5 μM) for 30 min, followed by incubation with ox-LDL (80 μg/ml) for 24 h. Samples were then collected for measurements(Figs 4–6).

### Protein sample preparation

Cells in 75 cm^2^ flask were prepared for protein samples. In brief, cells were washed with ice cold PBS for three times and harvested. Thereafter, cells were collected and re-suspended in lysis buffer (0.5 M triethylammonium bicarbonate (TEAB) and 1% sodium dodecyl sulphate) at 4 °C. The cell lysate was subjected to intermittent sonication using a Vibra Cell™ high-intensity ultrasonic processor (Jencon, Leighton Buzzard, Bedfordshire, UK). The remaining unbroken cells and debris were removed by centrifugation at 12,000 × *g* at 4 °C for 10 min. The protein concentrations of the cleared lysates were quantified using a 2-D Quant Kit (GE Healthcare). A standard curve was made using bovine serum albumin as a control.

### Protein digestion and iTRAQ labeling

Each sample was divided equally between two tubes. Proteins were first reduced with 5 mM tris-carboxyethyl phosphine hydrochloride for 1 h at 60 °C, alkylated with 10 mM methylethane thiosulphonate for 30 min at room temperature (RT) in the dark. The alkylated proteins were then diluted 10 times with deionized water prior to digestion with sequence-grade modified trypsin overnight at 37 °C (1:50 trypsin:sample), and then dried in a Speedvac (Thermo Electron, Waltham, MA, USA). The generated peptides were labeled with iTRAQ reagents, according to the manufacturer’s protocol (Applied Biosystems). Briefly, digested proteins were reconstituted in 30 μl of dissociation buffer (0.5 M TEAB) and mixed with 70 μl of ethanol-suspended iTRAQ reagents (one iTRAQ reporter tag per protein sample). The samples were labeled with the respective tags as follows: ox-LDL group = iTRAQ 115; HSYA treatment group = iTRAQ 116. Labeling reactions were carried out at RT for 1 h before all the samples were mixed into a single tube and dried using a Speedvac.

### Strong cation exchange fractionation

The combined iTRAQ-labeled samples were reconstituted with 200 μl buffer A (10 mM KH_2_PO_4_, pH 3.0, 25% v/v acetonitrile), and loaded onto a polysulphoethyl A column (200 mm length × 4.6 mm internal diameter, 200-Å pore size, 5 μm particle size) (PolyLC, MD, USA) in a prominence HPLC system (Shimadzu, Kyoto, Japan). The sample was isolated using a gradient of 100% buffer A for 5 min, 5%–30% buffer B (10 mM KH_2_PO_4_, pH 3.0, 500 mM KCl, and 25% v/v acetonitrile) for 40 min, 30%–100% buffer B for 5 min, and finally 100% buffer B for 5 min, at a constant flow rate of 1 ml/min for a total of 60 min. The eluted fractions were monitored through a UV detector at a wavelength of 214 nm. Fractions were collected at 1-min intervals and consecutive fractions with low peak intensity were combined. Finally, 20 fractions were obtained and dried in a Speedvac (Thermo Electron). Each fraction was reconstituted in 0.1% trifluoroacetic acid and desalted using a Sep-Pak C-18 SPE cartridge (Waters, Milford, MA, USA). Desalted samples were dried in a Speedvac (Thermo Electron) and stored at −20 °C prior to mass spectrometric analysis.

### Mass spectrometric analysis using Q-STAR

Each dried fraction was reconstituted in 100 μl of 0.1% formic acid and 2% acetonitrile and analysed multiple times using a Q-Star Elite mass spectrometer (MDS-Sciex; Applied Biosystems), coupled to a prominence HPLC system (Shimadzu). For each analysis, 100 µl of peptide mixture was injected in a Zorbax peptide trap (Agilent, CA, USA) and separated on a home-packed nanobored C18 column with a picofrit nanospray tip (75 μm ID × 15 cm, 5 µm particles) (New Objectives, Wubrun, MA, USA). The separation was performed at a constant flow rate of 0.3 µl/min with a 120 min gradient. The mass spectrometer was set to perform data acquisition in the positive ion mode, with a selected mass range of 300–2000 m/z. Peptides with +2 to +4 charge states were selected for MS/MS and the time of summation of MS/MS events was set to 2 s. The three most abundantly charged peptides above a five-count threshold were selected for MS/MS and dynamically excluded for 30 s with ± 30 mmu mass tolerance.

### Data analysis and interpretation

Peptides and proteins were identified using ProteinPilot™ Software (v3.0.0.0, Applied Biosystems) by searching against the Uniprot-Human database. The Paragon algorithm in ProteinPilot software was used whereby trypsin was selected as the digestion agent and cysteine modification of methylethane thiosulfonate. The search also allowed for the possibilities of more than 80 biological modifications using the BLOSUM 62 matrix. All reported proteins had at least two unique peptide matches with iTRAQ ratios, at least one of which must have an expectation value < 0.05. Finally, a concatenated target-decoy database search strategy was performed to estimate the rate of false positives, which was determined as < 1% for the current analysis. To account for small differences in protein loading, all protein ratios were normalized using the overall ratios for all proteins in the sample, as recommended by Applied Biosystems.

### Western blot analysis

Total protein lysates extracted from the samples as described above for LC-MS/MS analysis were used for western blotting to verify differentially expressed proteins. Cell samples from siRNA experiments were also collected and lysed for western blot analysis. In brief, total proteins were separated by sodium dodecyl sulphate-polyacrylamide gel electrophoresis and transferred onto nitrocellulose membranes. The membrane was blocked with 5% non-fat dry milk in TBST buffer (10 mmol/L Tris, 150 mmol/L NaCl, 0.05% Tween-20, pH 7.5) at RT for 1 h, and then hybridized with primary antibodies (see below, Supplementary Table S2) followed by infrared dye-conjugated secondary antibody (1:3000). After washing three times in TBST buffer, the membranes were visualized and analysed using an Odyssey infrared imaging system (LI-COR Biosciences, Lincoln, NE, USA). The following antibodies were used: Annexin V (ANXA5), GSTP1, HMG-17, VDAC2, and GAPDH. Four independent experiments were performed.

### Determination of MDA and NO concentrations

MDA and NO levels were measured in conditioned medium using assay kits, according to the manufacturers’ instructions. Briefly, MDA content was expressed as thiobarbituric acid reactive substance, measured by spectrophotometry, which reflected the level of lipid peroxidation. NO levels were determined indirectly as the contents of nitrite and nitrate; nitrite was converted to nitrate using aspergillus nitrite reductase, and total nitrate was measured using Griess reagent. The absorbance at 540 nm was determined by spectrophotometry.

### Administration of VDAC2 siRNA

Cells were transfected with VDAC2 siRNA(human) (sc-42357) (Santa Cruz Biotechnology) using a transfection reagent, according to the manufacturer’s instructions. Briefly, cells were plated into 6-well plates and cultured to confluence for 2 days. They were then pre-incubated for 15 min with siRNA transfection reagent (sc-29528) in siRNA transfection medium (sc-36868), and then transfected with VDAC2-specific or control siRNA. After 6 h of transfection, the medium was replaced with culture medium containing 10% fetal bovine serum. The VDAC2 content was determined by western blot analysis.

### TUNEL/DAPI and Annexin **V**/PI staining assays

Apoptosis, a form of cell death, is an important component in the progression of atherosclerosis. We studied cell death using two different standard methods (TUNEL/DAPI and Annexin V/PI). Annexin V-FITC and PI double staining was performed as described previously^48^. In brief, cultured EA.hy926 cells were washed with cold phosphate-buffered saline and binding buffer, followed by incubation with Annexin V-FITC for 10 min and PI staining for another 5 min at 4 °C in the dark. The stained cells were analysed by fluorescence microscopy within 30 min. DNA fragmentation and nucleus condensation in EA.hy926 cells was also examined by TUNEL-DAPI co-staining, as described previously^49^. Cells were fixed in 4% methanol-free formaldehyde in phosphate-buffered saline. After permeabilization, DNA strand breaks were labeled with fluorescein-12-dUTP, stained with DAPI, and analysed by fluorescence microscopy.

### Statistical analysis

Data were analysed by SPSS 14.0 software (SPSS Inc., Chicago,IL, USA) using one-way analysis of variance (ANOVA) with Student–Newman–Keuls post hoc test. All results are expressed as mean ± standard error. A *p* value < 0.05 was considered to be statistically significant.

### Data availability

Data generated or analysed during this study are included in this published article. Other data that may support the findings of this study are available from the corresponding author on reasonable request.

## Electronic supplementary material

Supplementary Tables

Supplementary Figure 1

Supplementary Figure 2

Supplementary Figure 3
