# Supplementary material for: Proteomic investigation of effects of hydroxysafflor yellow A in oxidized low-density lipoprotein-induced endothelial injury
Source: Sci Rep. 2017 Dec 21;7:17981. doi: 10.1038/s41598-017-18069-4 (PMC5740064; doi:10.1038/s41598-017-18069-4)
Supplement: Supplementary file 1 — Supplementary Tables [file 41598_2017_18069_MOESM1_ESM.pdf]

Proteomic investigation of effects of hydroxysafflor yellow A in oxidized low-density lipoprotein-induced endothelial injury

Feng Ye[1], Jianhe Wang[2],Wei Meng[3], Jingru Qian[3], and Ming Jin [1]*

Table Legends

Table S1. List of differential expressed proteins in HSYA treatment. *A 116*:115 is the ratio of different protein expression level in HSYA treatment compared to ox-LDL injury.

Table S2. Antibodies for western blot.

Table S1. iTRAQ analysis of differentially expressed proteins in the HSYA treatment relative to ox-LDL injury.

Number	Accession	Protein	116:115	Function
1	P16403	Histone H1.2（H12）	2.488857	Macromolecular structure. Signal transduction.
2	Q9BPW8	NipSnap homolog 1（NIPS1）	1.819701	Cellular_component.
3	P16402	Histone H1.3（H13）	1.786488	Macromolecular structure. Signal transduction.
4	P45880	Voltage-dependent anion-selective channel protein 2（VDAC2）	1.721869	Cellular structure. Forms a channel. Signal transduction.
5	Q53GU8	Transforming growth factor, beta-induced, 68kDa variant	1.706082	Molecular function unknown.
6	Q9BTM1	Histone H2A.J（H2AJ）	1.674943	Macromolecular structure. Core component of nucleosome. Signal transduction.
7	Q6PK18	2-oxoglutarate and iron-dependent oxygenase domain-containing protein 3（OGFD3）	1.659587	Dioxygenase Oxidoreductase.
8	Q9NPF4	Probable tRNA threonylcarbamoyladenosine biosynthesis protein（OSGEP）	1.644372	Component of the EKC/KEOPS complex.
9	Q8IVF7	Formin-like protein 3 （FMNL3）	1.599558	Plays a role in the regulation of cell morphology and cytoskeletal organization. Required in the control of cell shape and migration.
10	P10809	60 kDa heat shock protein, mitochondrial（CH60）	1.570363	Mitochondrial protein import and macromolecular assembly.
11	P20700	Lamin-B1（LMNB1）	1.570363	Components of the nuclear lamina, a fibrous layer on the nucleoplasmic side of the inner nuclear membrane.
12	Q567R6	Single-stranded DNA-binding protein	1.570363	Dioxygenase Oxidoreductase. Cellular_component. Single-stranded DNA binding.
13	Q5U4P5	GAK protein	1.5417	Kinase Transferase.
14	P16401	Histone H1.5（H15）	1.527566	Macromolecular structure. Signal transduction.
15	O95831	Apoptosis-inducing factor 1（AIFM1）	1.527566	Functions both as NADH oxidoreductase and as regulator of apoptosis.
16	Q9ULH0	Kinase D-interacting substrate of 220 kDa（KDIS）	1.527566	Signal transduction.
17	P23258	Tubulin gamma-1 chain（TBG1）	1.527566	Cellular structure(the major constituent of microtubules) .
18	P09417	Dihydropteridine reductase（DHPR）	1.527566	Catalytic activity, an essential cofactor for phenylalanine, tyrosine, and tryptophan hydroxylases.
19	O95810	Serum deprivation-response protein（SDPR）	1.513561	Intracellular trafficking.
20	Q8WWP7	GTPase IMAP family member 1（GIMA1）	1.513561	Immune function.
21	P22626	Heterogeneous nuclear ribonucleoproteins A2/B1（ROA2）	1.499685	Signal transduction.
22	P21796	Voltage-dependent anion-selective channel protein 1（VDAC1）	1.458814	Cellular structure. Forms a channel. Signal transduction.
23	P13804	Electron transfer flavoprotein subunit alpha, mitochondrial （ETFA）	1.458814	Carbohydrate metabolic process.

24	E9PI68	Signal peptidase complex subunit 2	1.458814	Cellular_component. Signal peptidase complex. Peptidase.
25	P07910-2	Isoform C1 of Heterogeneous nuclear ribonucleoproteins C1/C2（HNRPC）	1.44544	Signal transduction.
26	Q9BRX8	Redox-regulatory protein FAM213A（F213A）	1.44544	Metabolic process. Signal transduction.
27	B2RE46	FLJ96923, highly similar to ribophorin II (RPN2)	1.44544	Metabolic process. Signal transduction.
28	P41223	BUD31	1.44544	Signal transduction.
29	Q6NZ59	ATP synthase-coupling factor 6, mitochondrial	1.432188	Carbohydrate metabolic process.
30	P05204	Non-histone chromosomal protein HMG-17（HMGN2）	1.419057	Cellular_component.
31	O14818	Proteasome subunit alpha type-7（PSA7）	0.711214	Cellular_component. Signal transduction.
32	B0V043	Valine--tRNA ligase	0.711214	Cellular_component. Aminoacyl-tRNA editing activity. Regulation of translational fidelity.
33	B4DIE3	Protein disulfide-isomerase TMX3	0.711214	Metabolic process. isomerase. Protein disulfide oxidoreductase.
34	Q7KZ85	Transcription elongation factor SPT6（SPT6H）	0.711214	Signal transduction. Cellular_component.
35	P14618	Pyruvate kinase PKM（KPYM）	0.704693	Metabolic process. Signal transduction.
36	P62136	Serine/threonine-protein phosphatase PP1-alpha catalytic subunit（PP1A）	0.704693	Metabolic process. Signal transduction. Cellular_component.
37	P09211	Glutathione S-transferase P（GSTP1）	0.698232	Signal transduction.
38	Q06323	Proteasome activator complex subunit 1（PSME1）	0.691831	Immune function. Cellular_component.
39	Q53YD8	ADP-ribosylation factor-like 2	0.691831	Signal transduction. Cellular_component. Metabolic process.
40	P40227	T-complex protein 1 subunit zeta（TCPZ）	0.685488	Molecular chaperone.
41	O43707	Alpha-actinin-4（ACTN4）	0.679204	Bundling protein. Vesicular trafficking.
42	Q5TZZ9	Annexin（ANXA1）	0.679204	Immune function. Signal transduction.
43	P05787	Keratin, type II cytoskeletal 8（K2C8）	0.679204	Cellular_component.
44	O60701	UDP-glucose 6-dehydrogenase（UGDH）	0.679204	Metabolic process.
45	Q13813	Spectrin alpha chain, non-erythrocytic 1（SPTN1）	0.672977	Secretion.
46	E5RGS2	Receptor expression-enhancing protein 4	0.672977	Molecular function unknown.
47	B2R9M7	FLJ94469, highly similar to protein kinase C, epsilon (PRKCE)	0.666807	Signal transduction.
48	Q9NPA8	Enhancer of yellow 2 transcription factor homolog（ENY2）	0.654636	Signal transduction. Subunit structure.
49	Q86T13	C-type lectin domain family 14 member A（CLC14）	0.654636	Cellular_component. Carbohydrate binding.
50	E9PDN5	Dystrophin	0.654636	Calcium ion binding. Zinc ion binding.
51	P46109	Crk-like protein（CRKL）	0.642688	Signal transduction. Development. Organ morphogenesis.
52	P12814	Alpha-actinin-1（ACTN1）	0.636796	Bundling protein.

53	P62277	40S ribosomal protein S13（RS13）	0.636796	Ribonucleoprotein. Ribosomal protein.
54	O75436	Vacuolar protein sorting-associated protein 26A（VP26A）	0.625173	Subunit structure (Essential component of the retromer complex). Signal transduction
55	Q6P452	Annexin（ANXA4）	0.619441	Calcium ion binding. Calcium-dependent phospholipid binding.
56	Q15369	Transcription elongation factor B polypeptide 1（ELOC）	0.613762	Signal transduction. Subunit structure. Proteasomal degradation of target proteins.
57	Q9Y2D5	A-kinase anchor protein 2（AKAP2）	0.60256	Signal transduction.
58	P78417	Glutathione S-transferase omega-1（GSTO1）	0.60256	Catalytic activity.
59	Q96IH1	Fascin	0.591562	Cellular_component. Actin filament bundle assembly
60	Q5W0H4	Translationally-controlled tumor protein	0.591562	Molecular function unknown.
61	Q9NXR1	Nuclear distribution protein nudE homolog 1（NDE1）	0.591562	Development. Proliferation. Signal transduction.
62	Q9UHY7	Enolase-phosphatase E1（ENOPH）	0.586138	Catalytic activity.
63	Q9UI42	Carboxypeptidase A4（CBPA4）	0.559758	Signal transduction. Metalloprotease.
64	Q8N7G1	Purine nucleoside phosphorylase	0.549541	Catalytic activity. Purine nucleoside phosphorylase.
65	P07355	Annexin A2	0.534564	Cellular_component. Calcium signal. May be involved in heat-stress response.
C	P08758	Annexin A5	0.534564	Anticoagulant protein.
67	Q96I24	Far upstream element-binding protein 3(FUBP3)	0.524808	Signal transduction. May interact with single-stranded DNA from the far-upstream element (FUSE).
68	Q9NX40	OCIA domain-containing protein 1(OCAD1)	0.524808	Cellular_component.
69	P21108	Ribose-phosphate pyrophosphokinase 3 (PRPS3)	0.515229	Catalytic activity.
70	Q71DI3	Histone H3.2 (H32)	0.487529	Macromolecular structure. Signal transduction.
71	P07900	Heat shock protein HSP 90-alpha (HS90A)	0.483059	Molecular chaperone.
72	Q6FHM2	GNB2 protein	0.47863	Cellular_component. Signal transduction. Calcium channel regulator activity.
73	Q5SRT3	Chloride intracellular channel 1, isoform CRA_a	0.474242	Signal transduction. Cellular_component.
74	Q9NSI2	FAM207A	0.457088	Molecular function unknown.
75	O60826	Coiled-coil domain-containing protein 22(CCD22)	0.420727	Subunit structure. Interacts with CPNE1 and CPNE4.
76	Q9Y6E3	HSPC027	0.383707	Molecular function unknown.
77	P62805	Histone H4	0.235505	Macromolecular structure. Signal transduction.

Table S2. Antibodies for western blot

Name	Gene name	Cat No	Company
Glutathione S-transferase P	GSTP1	sc-66000	Santa Cruz Biotechnology
The high-mobility group protein 17	HMG-17	sc-19073	Santa Cruz Biotechnology
Annexin V	ANXA5	sc-8300	Santa Cruz Biotechnology
Glyceraldehyde-3-phosphate dehydrogenase	GAPDH	AF0006	Beyotime Biotechnology
Voltage-dependent anion-selective channel protein 2	VDAC2	9412	Cell Signaling Technology
