# Supplementary material for: Proteomic investigation of effects of hydroxysafflor yellow A in oxidized low-density lipoprotein-induced endothelial injury
Source: Sci Rep. 2017 Dec 21;7:17981. doi: 10.1038/s41598-017-18069-4 (PMC5740064; doi:10.1038/s41598-017-18069-4)
Supplement: Supplementary file 2 — Supplementary Figure 1 [file 41598_2017_18069_MOESM2_ESM.doc]

**Proteomic investigation of effects of hydroxysafflor yellow A in oxidized low-density lipoprotein-induced endothelial injury**

Feng Ye^1^, Jianhe Wang^2^,Wei Meng^3^, Jingru Qian^3^, and Ming Jin ^1^*

**
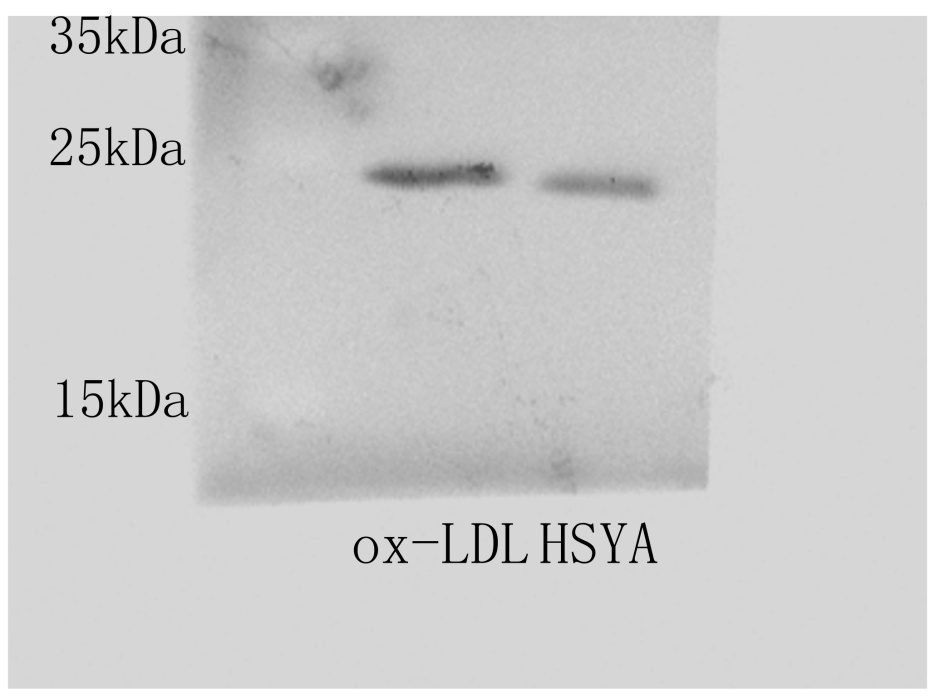
**

Supplementary Figure 1.a: GSTP1


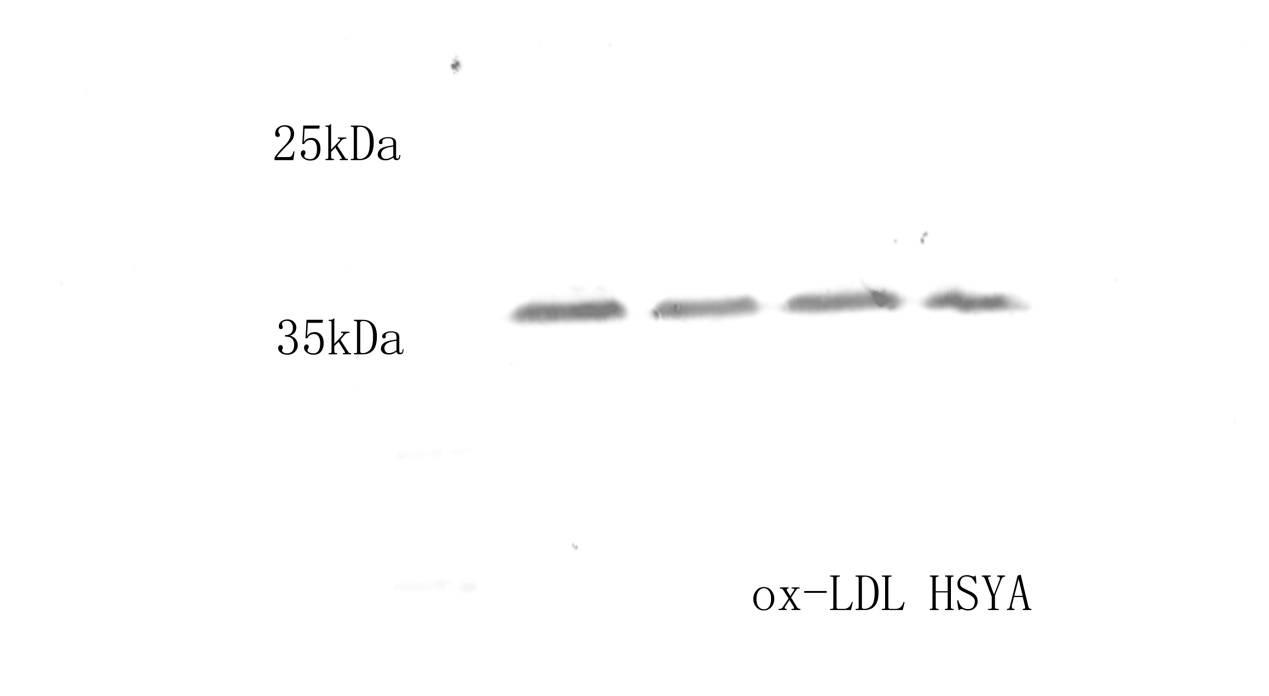


Supplementary Figure 1.b: ANXA5


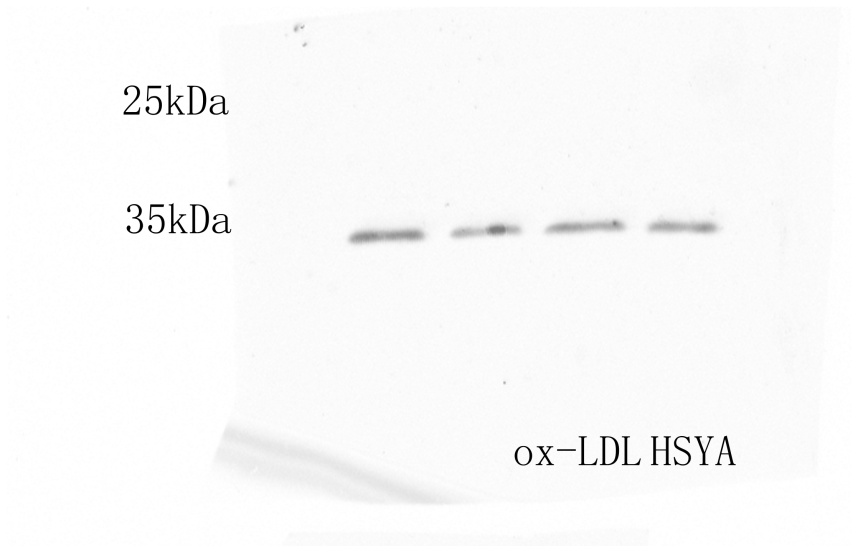


Supplementary Figure 1.c: GAPDH


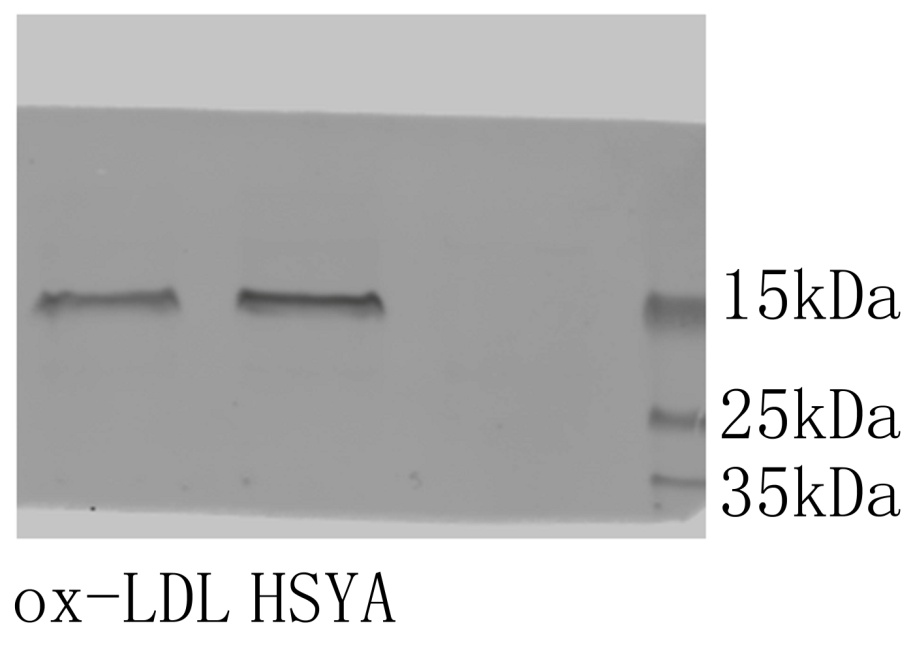


Supplementary Figure 1.d: HMG-17


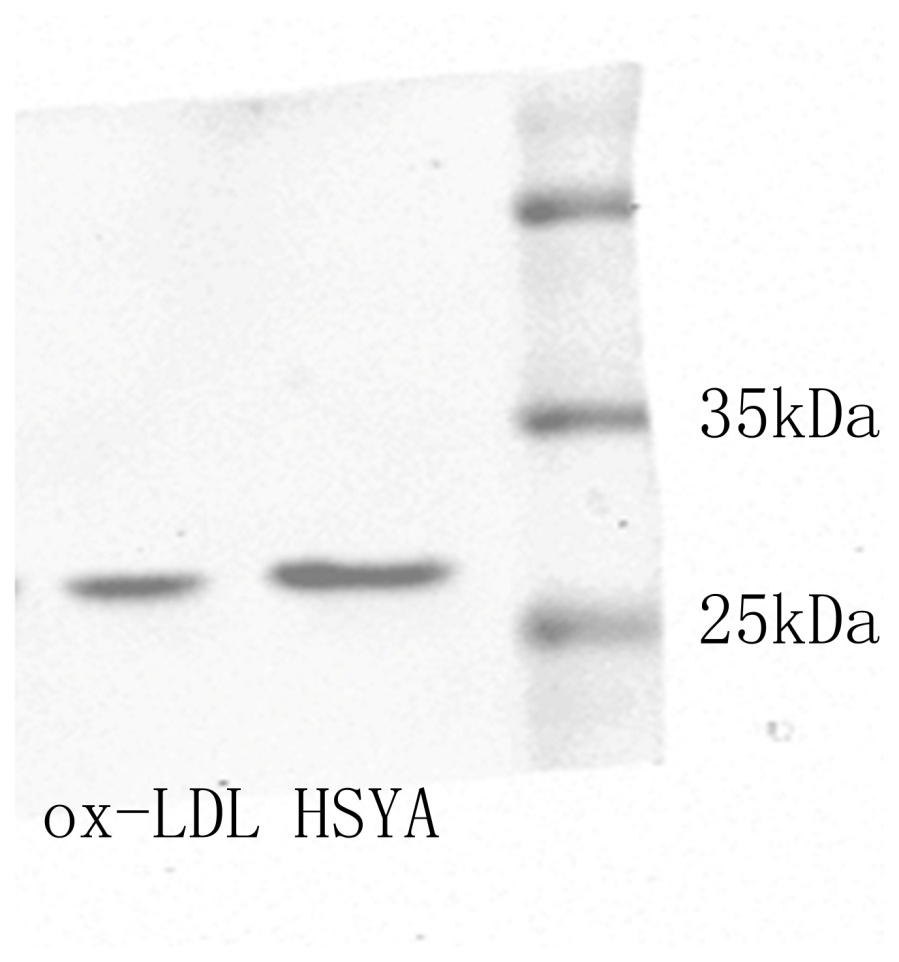


Supplementary Figure 1.e: VDAC2
