# Supplementary material for: Proteomic investigation of effects of hydroxysafflor yellow A in oxidized low-density lipoprotein-induced endothelial injury
Source: Sci Rep. 2017 Dec 21;7:17981. doi: 10.1038/s41598-017-18069-4 (PMC5740064; doi:10.1038/s41598-017-18069-4)
Supplement: Supplementary file 4 — Supplementary Figure 3 [file 41598_2017_18069_MOESM4_ESM.doc]

**Proteomic investigation of effects of hydroxysafflor yellow A in oxidized low-density lipoprotein-induced endothelial injury**

Feng Ye^1^, Jianhe Wang^2^,Wei Meng^3^, Jingru Qian^3^, and Ming Jin ^1^*


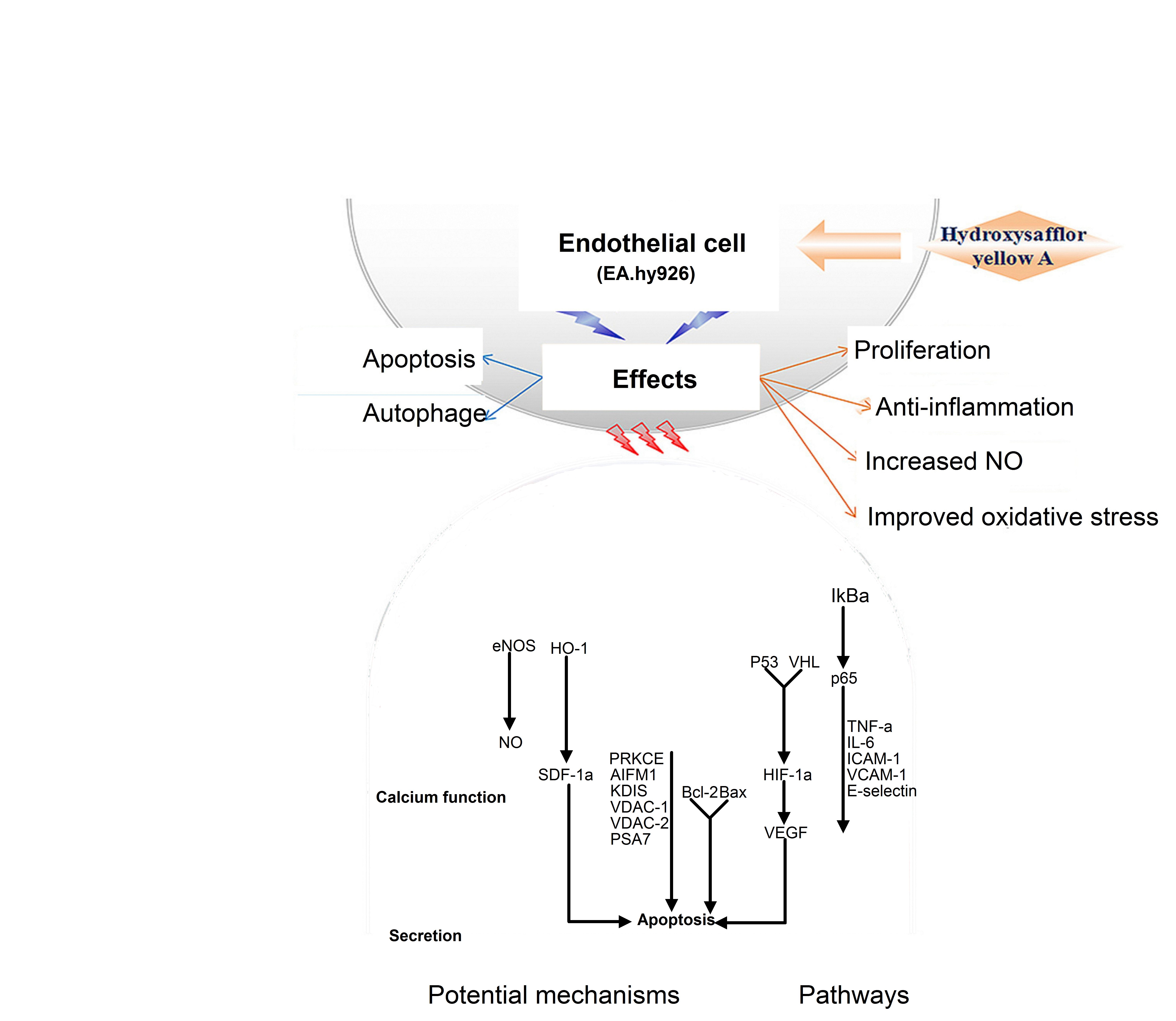


Supplementary Figure 3.
